# Nerve transfer of the teres minor motor branch to the long head of the triceps muscle in C6–T1 brachial plexus palsy

**DOI:** 10.1177/17531934251337550

**Published:** 2025-05-05

**Authors:** Sébastien Durand, Daniel Estoppey, Julie Mercier

**Affiliations:** Department of Hand Surgery, Lausanne University Hospital and University of Lausanne, Switzerland

**Keywords:** Axillary nerve, brachial plexus palsy, long head of the triceps, shear wave elastography, teres minor

## Abstract

We report a case of restoration of elbow extension after C6–T1 brachial plexus injury using nerve transfer of the teres minor motor branch to the long head of the triceps muscle.

**Level of evidence:** V

Approximately 1% of brachial plexus palsies occur after C6–T1 injury with shoulder motion and elbow flexion often preserved while elbow extension is typically lost owing to triceps paralysis. Reconstruction options include posterior deltoid transfer to the triceps using a fascia lata graft; however, if surgery is carried out within 6 months of injury, nerve transfers can be a more physiological option (Bertelli and Ghizoni, 2011). Transfer of the motor branch of the teres minor muscle to the long head of biceps muscle has been described before to restore elbow extension in a tetraplegic patient (Bertelli et al., 2011a). We report a case of restoration of elbow extension using nerve transfer of the teres minor motor branch to the long head of the triceps muscle in a 22-year-old man (Video S1).

He sustained a partial right brachial plexus injury (C6–T1) after a motorcycle accident. The hand, elbow flexion and extension were paralysed but the deltoid, teres minor, supra- and infraspinatus muscles were functional. MRI confirmed avulsion of the C8–T1 roots and revealed thickening and oedema of the remaining brachial plexus. Nerve transfer of the teres minor muscle to the long head of the triceps muscle and transfer of the third, fourth and fifth intercostal nerves to the nerve of the biceps muscle was carried out 6 months after the injury. Through a thoraco-axillary approach the latissimus dorsi tendon was retracted to expose the quadrangular space. The anterior and posterior divisions of the axillary nerve were identified, and intraoperative electrical stimulation confirmed the identity of all nerve branches, together with the branch to the teres minor and posterior deltoid muscles. A neuroma of the musculocutaneous nerve was observed proximally in the coraco-brachial muscle. The teres minor motor branch was freed and divided as distally as possible. The triceps long head motor branch was identified, dissected as proximally as possible, divided and sutured to the branch of the teres minor (Figure 1). The transfer of the third, fourth and fifth intercostal nerves to the nerve of the biceps muscle without interpositional nerve graft was carried out (Cho et al., 2015). All the nerve sutures were inserted under the microscope with 10–0 ethilon (Ethicon Inc., Somerville, NJ, USA) and surrounded by a ﬁbrin sealant sheath.

**Figure 1.**
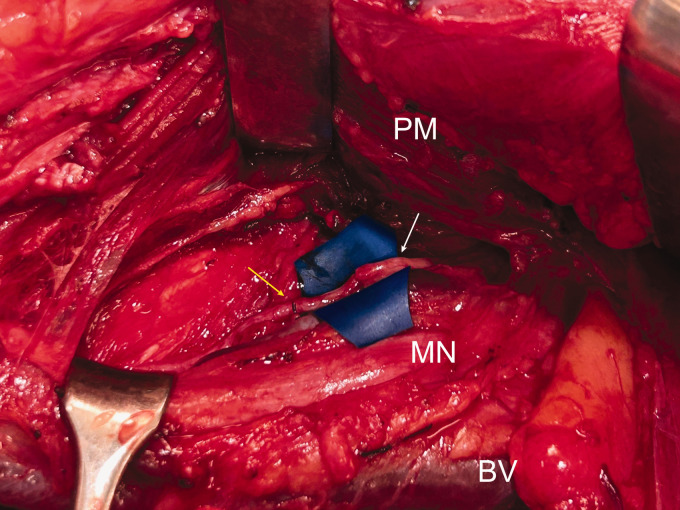
Intraoperative view showing the transfer of the nerve of the teres minor muscle (white arrow) to the nerve of the long head of the triceps muscle (yellow arrow). Note the close match between the diameters of these branches. PM, Pectoralis major muscle; MN, median nerve; BV, brachial vein.

At 12 months follow-up, active elbow flexion was 130° (M3+ according to Medical Research Council scoring) and active elbow extension was complete (M3+) (Figure 2(a) and 2(b)). Shear wave elastography was carried out using the Aixplorer™ ultrasound system (Supersonic Imagine, Aix-en-Provence, France). The shear wave modulus of the long head of the triceps and biceps (Figure 2(c)–2(f)) increased distinctly from rest to active motion correlating with restoration of contraction.

**Figure 2.**
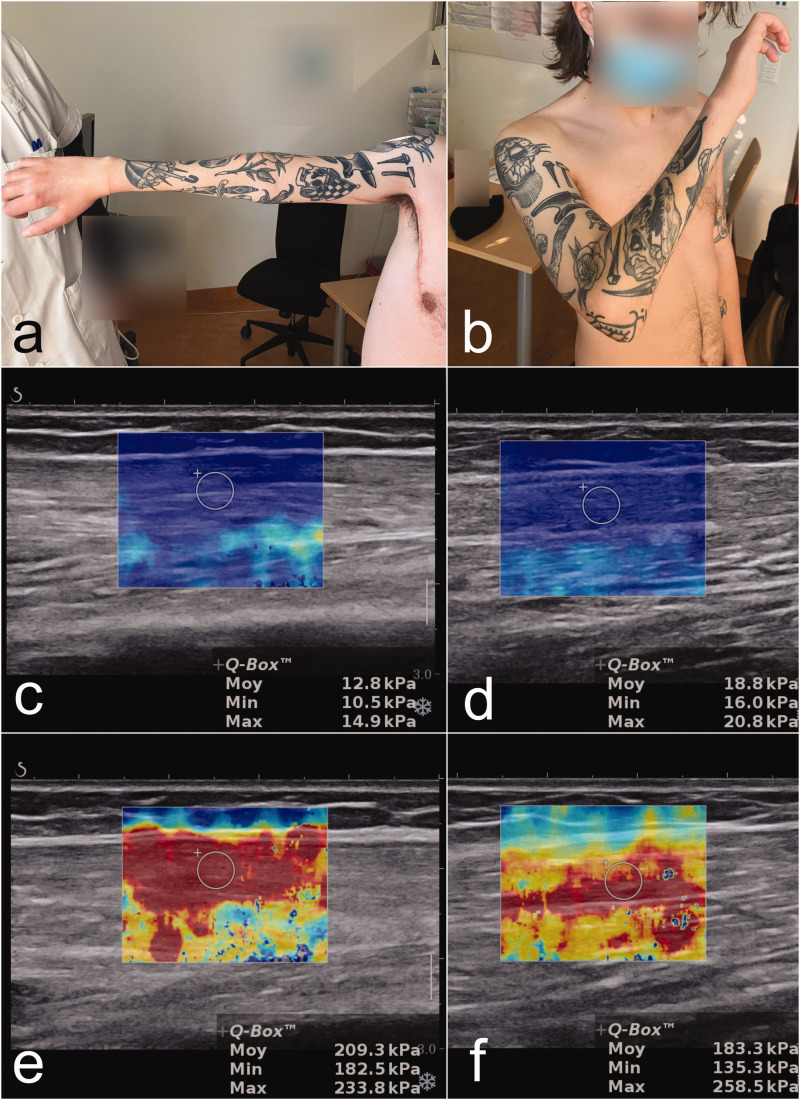
Results of elbow extension and flexion on the right side 12 months after surgery (a and b). Shear wave elastography shows an increase of the shear wave modulus of the long head of the triceps muscle from 12.8 kPa at rest (c) to 209.3 kPa during active extension (e) and for the biceps muscle from 18.8 kPa at rest (d) to 183.3 kPa during active elbow flexion (f). At rest the muscle is soft (low elastographic values, coloured in blue) but when contracted, the muscle becomes taunt and stiff (higher elastographic values, coloured in orange/red).

The axillary nerve, that carries nerve fibers from C5 and C6, emerges from the upper portion of the posterior cord and divides into anterior and posterior branches. The posterior branch consistently sends a branch to the teres minor muscle proximally and a branch to the posterior deltoid muscle distally and their diameters and the number of myelinated fibres are a close match (Bertelli et al., 2011b). The branch of the teres minor muscle can be transferred tension-free directly to the long head of the triceps muscle and our case shows that elbow extension in C6–T1 brachial plexus injuries can be restored.

## Supplemental Material

sj-mp4-1-jhs-10.1177_17531934251337550 - Supplemental material for Nerve transfer of the teres minor motor branch to the long head of the triceps muscle in C6–T1 brachial plexus palsy Supplemental material, sj-mp4-1-jhs-10.1177_17531934251337550 for Nerve transfer of the teres minor motor branch to the long head of the triceps muscle in C6–T1 brachial plexus palsy by Sébastien Durand, Daniel Estoppey and Julie Mercier in Journal of Hand Surgery (European Volume)
